# SST interneuron maturation extends beyond the second postnatal week and is driven by coordinated transcriptional and functional remodeling

**DOI:** 10.3389/fnmol.2026.1892850

**Published:** 2026-09-15

**Authors:** Ourania Christodoulou, Konstantinos Diskos, Angeliki Velli, Panagiotis Moulos, Kyriaki Sidiropoulou, Myrto Denaxa

**Affiliations:** 1 Department of Biology, School of Sciences and Engineering, University of Crete, Heraklion, Greece; 2 Biomedical Sciences Research Center “Alexander Fleming”, Vari, Greece; 3 Institute of Molecular Biology and Biotechnology, Foundation for Research and Technology Hellas, Heraklion, Greece; 4 Department of Physiology, School of Medicine, University of Patras, Patras, Greece

**Keywords:** cortical interneurons, GABA receptor subunits, glutamate receptor subunits, postnatal development, somatostatin, voltage-gated channels

## Abstract

Cortical somatostatin-expressing (SST+) interneurons (cINs) are key regulators of dendritic integration and synaptic plasticity, yet the timing and molecular mechanisms underlying their postnatal maturation remain incompletely understood. Previous studies have suggested that key aspects of SST+ cIN maturation are largely established by the end of the second postnatal week. Here, we combined transcriptomic profiling and electrophysiological recordings to characterize the developmental trajectory of SST+ interneurons in the mouse neocortex from the second postnatal week through the end of the first postnatal month. Bulk RNA sequencing of fluorescence-activated cell sorted SST+ cINs at P10, P15, and P30 revealed extensive transcriptional remodeling, including continued upregulation of genes associated with ion channel activity, synaptic transmission, and neuronal excitability beyond the second postnatal week. Consistent with these molecular changes, electrophysiological recordings demonstrated ongoing maturation of intrinsic and synaptic properties between P15 and P30. Notably, developmental upregulation of voltage-gated sodium and potassium genes correlated with the observed changes in intrinsic excitability, while alterations in GABA_*A*_ receptor subunit expression paralleled the maturation of inhibitory synaptic transmission. Computational modeling incorporating these transcriptional changes recapitulated the electrophysiological differences between P15 and P30 SST+ cINs. Together, our findings demonstrate that SST+ cIN maturation is not complete by the second postnatal week but instead continues through later postnatal stages, revealing a prolonged and coordinated transcriptional and physiological program underlying their functional integration into cortical circuits.

## Introduction

Cortical GABAergic interneurons (cINs), the primary inhibitory neurons of the cortex, comprise a diverse range of neurons, which can be subdivided into distinct subtypes based on their morphology, synaptic connectivity, electrophysiology and gene expression profile (Markram et al., 2004; Kepecs and Fishell, 2014; Tremblay et al., 2016). Recent studies suggest that while cIN fate is broadly determined when they are born, cINs acquire their complex features only postnatally, when they reach the cortex and integrate into diverse cortical circuits (Lim et al., 2018). It has been proposed that postnatal activity-regulated transcriptional programs operating downstream of early induced gene networks mediate the specific aspects of cIN maturation (Wamsley and Fishell, 2017). However, detailed molecular and physiological descriptions of the postnatal development of different cIN types are still underrepresented. Such knowledge is important for elucidating the specific molecular programs that mediate distinct mature cIN phenotypes. We address this question for one cIN subtype, the somatostatin-expressing (SST+) cINs.

Mature SST+ cINs control synaptic integration in pyramidal neurons, primarily by preferentially targeting their distal dendrites, and recent studies highlight their unique roles in brain function, including calcium-dependent dendritic plasticity (Furutachi et al., 2024) and their implication in some forms of learning (Adler et al., 2019; Dobrzanski et al., 2022). Most SST+ cINs are born in the medial ganglionic eminence (MGE) of the basal forebrain (Miyoshi et al., 2007) and comprise one of the three super-families of cINs, the other two being the parvalbumin- (PV) and the serotonin ionotropic receptor (5HTR3a)-expressing cINs (Rudy et al., 2011). Emerging evidence suggests that SST+ cINs are synaptically integrated in transient early circuits in the hippocampus and the neocortex during the first week of postnatal development, and co-ordinate activity across neonatal circuits (Bonifazi et al., 2009; Tuncdemir et al., 2016; Marques-Smith et al., 2016; Wang et al., 2019). However, during these early network events, SST+ cINs have hardly acquired their mature properties. Indeed, recent work has shown that the intrinsic and synaptic properties of SST+ cINs in the anterior cingulate cortex and other cortical regions largely mature at the end of the 2nd postnatal week (Lazarus et al., 2011; Kinnischtzke et al., 2012; Pan et al., 2016, 2019). The transcriptional programs underlying the evolving changes in SST+ physiology have not been fully elucidated.

We characterized both whole transcriptome expression and electrophysiology of SST+ cINs at key postnatal stages and found that SST+ cINs exhibit profound developmental regulation of both gene expression and physiology over the entire first month after birth. Comparing the temporal patterns of gene expression with the maturational changes in SST+ cINs physiological features, we correlated the expression of specific genes with the emergence of certain electrophysiological properties. These correlations have been further supported by a biophysically-detailed neuron model. By integrating transcriptomic, electrophysiological, and computational analyses across defined postnatal stages, our study reveals that SST+ cIN maturation extends well beyond the second postnatal week, elaborating on existing views of early maturation and establishing a direct link between ongoing gene expression programs and the emergence of their mature functional properties within cortical circuits.

## Materials and methods

### Animals

Sst-IRES-Cre (Taniguchi et al., 2011; JAX stock #013044; STOCK Ssttm2.1(cre)Zjh/J), which is expressed in SST+ neurons from early embryonic stages (Embryonic day (E) E13.5, Figures 5F, G in Taniguchi et al., 2011), Rosa26-tdTomato (Ai14 RFP; Madisen et al., 2010; JAX stock #: 007914; B6.Cg-Gt(ROSA)26Sortm14(CAG-tdTomato)Hze/J) and Rosa26-EYFP (R26R-EYFP; Srinivas et al., 2001; JAX stock #: 006148; B6.129 × 1-Gt(ROSA)26Sortm1(EYFP)Cos/J) mouse lines were kept on a C57BL/6 background. Both male and female mice were used in experiments. All mice were housed either at the BSRC Alexander Fleming Animal Facility or at the IMBB-FORTH Animal Facility according to the guidelines of the Institutional Animal Care and Use Committee of BSRC Alexander Fleming and IMBB FORTH, approved by the Veterinarian Authorities Office (protocol license no. 781431, no. 93164, no. 265179).

### Tissue dissociation and cell sorting

All mice were sacrificed by cervical dislocation -except for P10 pups that were euthanized by decapitation- and the whole cortex was dissected and isolated. Tissue dissociation was performed according to Saxena et al., 2012. Fluorescently labeled cells were sorted in FACS AriaIII, collected into RNase-free tubes containing Trizol LS (Invitrogen, 10296010), and stored at −80 °C.

### RNA sequencing and data analysis

Total RNA was extracted from sorted SST+ cIN samples using the PureLink™ RNA Micro Kit (Invitrogen, 12183-016) according to manufacturer’s recommendations. Libraries were constructed with the “QuantSeq 3′ mRNA-Seq LibraryPrepKit for IonTorrent” (Lexogen) and were subsequently sequenced on a IonTorrent Proton™ Next Generation Sequencer (ThermoFisher Scientific). Three biological replicates were used for each dataset. RNA-seq analysis was performed with the Bioconductor package metaseqR2. Differential gene expression analysis was performed with the DESeq2 algorithm (Fanidis and Moulos, 2021). Genes presenting an FDR value less than 0.05 and fold change greater than 1.0 or less than −1.0 in log_2_ scale were considered differentially expressed. Functional enrichment analyses were performed using three complementary approaches. Gene Set Enrichment Analysis (GSEA) was performed using the WebGestalt (WEB-based Gene SeT AnaLysis Toolkit) web server (Liao et al., 2019). A ranked gene list based on log2 fold change values obtained from the differential expression analysis was used as input for GSEA. Analyses were performed against the Gene Ontology (GO) Biological Process (non-redundant) database. Redundancy among enriched GO terms was reduced using the Weighted Set Cover (fast) algorithm with the mean collapse method. The minimum and maximum gene set sizes were set to 5 and 2000 genes, respectively, and enrichment significance was estimated using 1,000 permutations. The top enriched GO categories following redundancy reduction were retained for visualization. To complement the genome-wide GSEA, Gene Ontology (GO) overrepresentation analyses were performed using GeneCodis 4 (Garcia-Moreno et al., 2022) and the PANTHER Classification System (Mi and Thomas, 2009; Thomas et al., 2022). For both analyses, only differentially expressed genes meeting the thresholds of false discovery rate (FDR) < 0.05 and absolute log2 fold change (| log2FC|) ≥ 1 were included. In GeneCodis, enrichment analysis was performed using the hypergeometric test, the annotated gene universe as the reference background, and the Gene Ontology Biological Process annotation database, while all remaining parameters were left at their default settings. In PANTHER, statistical overrepresentation analysis was performed using the GO Biological Process Complete annotation dataset with the Mus musculus reference gene list. Statistical significance was assessed using Fisher’s exact test, and false discovery rate (FDR) correction was applied to account for multiple testing (only for results in Supplementary Figures 1D,E). All functional enrichment analyses were performed independently for each pairwise differential expression comparison. For the heatmaps, genes were selected from curated gene sets based on QIAGEN RT^2^ Profiler PCR Arrays (Neurogenesis and Neural Stem Cells, Apoptosis, Synaptic Plasticity, and Ion Channels and Transporters, Supplementary Table 1). Heatmaps include the subset of genes from these curated panels that were differentially expressed in the RNA-seq dataset (only differentially expressed genes meeting the thresholds of false discovery rate (FDR) < 0.05 and absolute log2 fold change (| log2FC|) ≥ 1 at least for one of two comparisons were included). Heatmaps were drawn with the Bioconductor package heatmap2.

### *Ex vivo* patch-clamp recordings

#### Slice preparation

Mice were decapitated under isoflurane anesthesia. The brain was removed immediately and coronal slices of the neocortex (300 μm thick), (Leica, VT1000S, Leica Biosystems) were prepared in ice-cold oxygenated (95% O_2_ - 5% CO_2_) choline-based aCSF (in mM): 125 choline-Cl, 2.5 mM KCl, 1.26 NaH_2_PO_4_, 0.5 CaCl_2_, 7 mM MgSO_4_, 26 NaHCO3, 5 glucose, pH = 7.4, 315 mOsm/l - as previously described (Kalemaki et al., 2022). Slices were allowed to equilibrate for at least 30 min at RT before being transferred to the recording chamber. During recordings, slices were perfused at a rate of 4 mL/min with continuously aerated (95% O2–5% CO2) normal aCSF at RT. The maintenance and recording aCSF contained (in mM): 126 NaCl, 3.5 KCl, 1.2 NaH_2_PO_4_, 26 NaHCO_3_, 1.3 MgCl_2_, 2.0 CaCl_2_, and 10 D-glucose, pH 7.4, 315 mOsm/l.

#### Data acquisition and analysis

Neurons were impaled with patch pipettes (5–7 MΩ) and recorded in the whole-cell configuration, either in the current-clamp or in the voltage-clamp mode, as previously described (Kalemaki et al., 2022). Data was analyzed using custom-written codes in IgorPro software (Wavemetrics, Inc.). For passive membrane properties, the resting membrane potential (RMP, mV) was measured within 3 min after establishing the whole-cell configuration and monitored throughout the experiment. To measure input resistance, a 500-ms step-pulse protocol was used with current stimulation from −200 to +50 pA. The input resistance (Rin, MΩ) was measured by plotting the steady-state voltage deflection in an I–V plot and calculating the slope of the best fit line curve (Rin = V/I). The active properties were measured at the minimum current stimulation that generated an AP. The AP threshold (mV) was calculated by taking the first derivative of the voltage trace, defining a threshold and identifying the voltage level at that time point. The rate of rise of the AP (dV/dt, mV/ms) was the maximum value of that first derivative trace. The AP amplitude (mV) was defined as the voltage difference between AP threshold and AP peak. The AP duration (ms) was calculated by the full width of the waveform at the half maximal amplitude (half-width). In the voltage-clamp recordings, the measurements of spontaneous excitatory post-synaptic currents (sEPSCs) were taken at –60 mV and for spontaneous inhibitory postsynaptic currents (sIPSCs) at +10 mV. We measured the number of sIPSC and sEPSC events per 5 s traces (for 10 consecutive traces) to measure the frequency of sIPSCs and sEPSCs. The frequency of 10 different traces was averaged. The amplitude was measured for all sIPSCs and sEPSCs and averaged.

#### Computational neuronal model

For modeling the electrophysiological properties of SST neurons, we used the code from Konstantoudaki et al., 2014. The SST neuron was modeled with a somatic, dendritic and an axonal compartment. The model was implemented in the NEURON simulation environment [ Hines and Carnevale, 2001 and simulations were executed on a xeon cluster (8-core processors)]. For the simulation, current was injected into the somatic compartment for 500 ms, and the voltage traces were analyzed. To reproduce the electrophysiological data recorded from SST interneurons, the biophysical conductances of the P15 and P30 model neurons used are shown in Table 2.

### Statistical analysis

Statistical analysis was performed in Microsoft Office Excel 365 and GraphPad Prism Software 7.0. Data are presented as mean standard error of mean ( ± SEM). Normality of the datasets was assessed with the Kolmogorov-Smirnov test. Comparisons between P30 and P15 were performed using *t*-test. The null hypothesis was rejected for a <0.05. Significance levels are indicated as *P < 0.05, **P < 0.01, ***P < 0.001.

## Results

### Transcriptomic profiling of SST+ cINs during later postnatal maturation

To profile the developmental transcriptome of SST+ cINs, we combined the Sst-Cre driver (Taniguchi et al., 2011) with the Cre-dependent fluorescent reporter Rosa26-tdTomato (tdT, Ai14, Madisen et al., 2010), and we FACS-sorted tdT+ SST-expressing cINs for bulk RNA-seq analysis, at three developmental stages: (1) at P10, when SST+ cIN cell death is concluded (Southwell et al., 2012; Wong et al., 2019), (2) at P15, when SST+ cINs are synaptically integrating into cortical networks, and (3) at P30 when the establishment of the cortical circuitry is well-advanced.

Transcriptomic analysis of P15 versus P10 SST+ cINs identified 2,659 differentially expressed genes (DEGs), of which 1,966 were downregulated and 693 were upregulated [false discovery rate (FDR) threshold of 0.05 and absolute fold change (FC) cutoff value of 1.0 in log_2_ scale] (Figure 1A). In comparison, analysis of P30 vs. P15 SST+ cINs identified 1,316 DEGs, of which 457 were downregulated and 859 were upregulated (Figure 1B).

**FIGURE 1:**
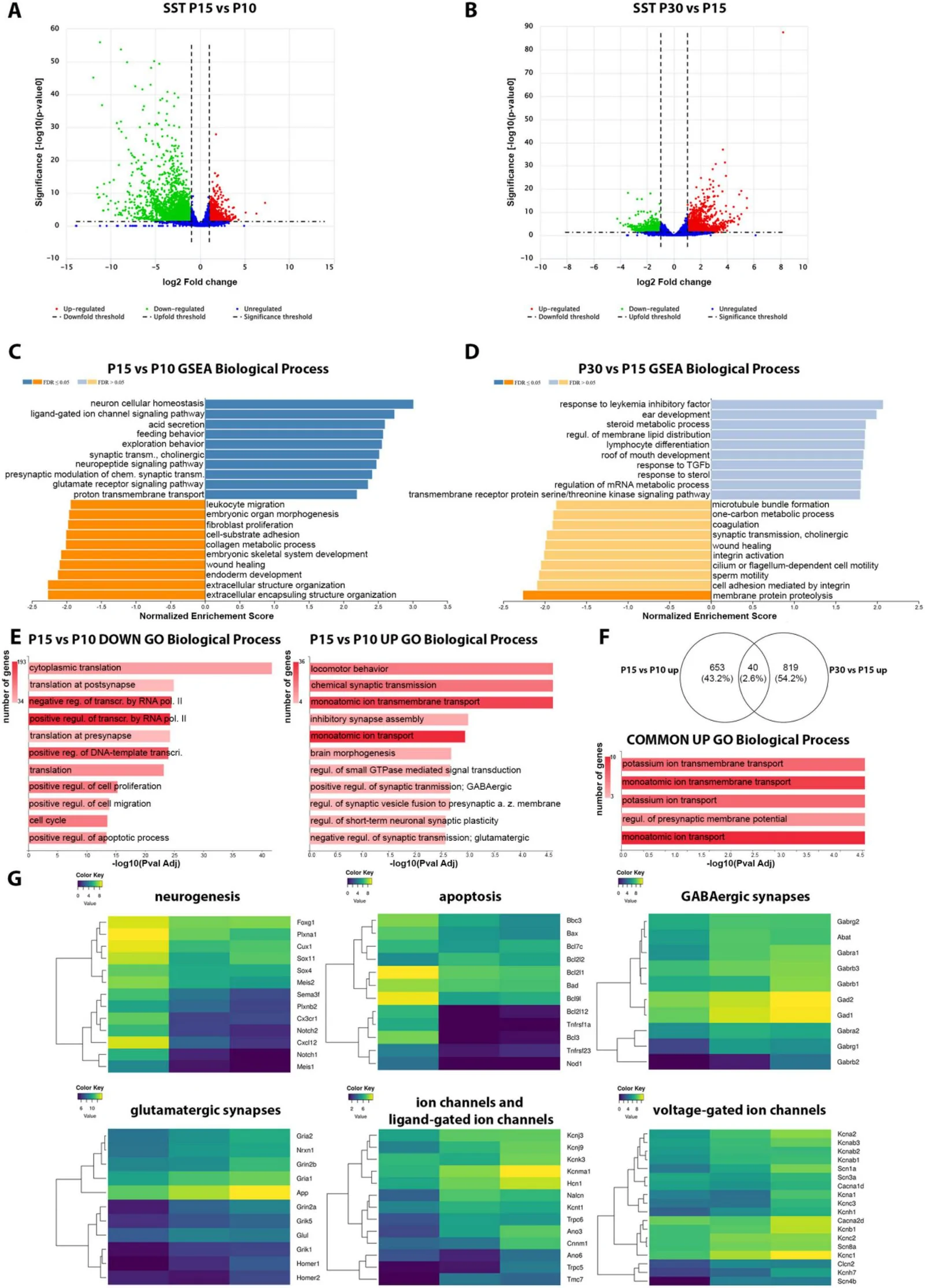
Transcriptomic analysis of the SST+ cortical interneurons during later postnatal maturation. **(A,B)** Volcano plots showing differentially expressed genes (DEGs) identified by bulk RNA sequencing of FACS-isolated SST+ cortical interneurons (cINs) from SST-Cre;Rosa26tdTomato mice. Comparisons were performed between P15 and P10 **(A)** and P30 and P15 **(B).** Red and green dots represent significantly upregulated (red) and downregulated (green) genes. Blue dots represent genes that did not meet the differential expression criteria (FDR ≤ 0.05 and |log2 fold change| ≥ 1). **(C,D)** Gene Set Enrichment Analysis (GSEA) of Biological Process gene sets. Normalized enrichment scores (NES) for significantly enriched pathways are shown for the P15 vs. P10 comparison **(C)** and the P30 vs. P15 comparison **(D)**. Positively enriched pathways are shown in blue and negatively enriched pathways are shown in orange. Dark colors indicate significantly enriched pathways with FDR ≤ 0.05, whereas lighter colors indicate pathways with FDR > 0.05. **(E)** Gene Ontology (GO) Biological Process over-representation analysis of DEGs (FDR ≤ 0.05 and |log2 fold change| ≥ 1) identified in the P15 vs. P10 comparison. Left, representative biological processes most significantly enriched among downregulated genes. Right, representative biological processes most significantly enriched among upregulated genes. Bars represent -log10 (adjusted *P*-value). **(F)** Top, Venn diagram showing overlap between genes upregulated in the P15 vs. P10 and the P30 vs. P15 comparisons. Bottom, GO Biological Process over-representation analysis of the 40 commonly upregulated genes. **(G)** Heatmaps display normalized transcript abundance (Z–score) of representative DEGs across P10, P15 and P30 developmental stages. Genes were selected from predefined, functionally curated gene sets (Supplementary Table 1) representing neurogenesis, apoptosis, GABAergic synapses, glutamatergic synapses, ion channels and ligand-gated ion channels and voltage-gated ion channels. Colors indicate expression relative to the mean expression of each gene across all samples. All transcripts included in the heatmaps have FDR cutoff 0.05 and absolute log_2_ fold-change cutoff 1. *N* = 3 biological replicates for each developmental time-point.

To identify the biological processes associated with these transcriptional changes, we first performed Gene Set Enrichment Analysis (GSEA). Comparison of P15 vs. P10 revealed significant changes in several biological processes. Negatively enriched pathways were primarily associated with early developmental programs including embryonic organ development, endoderm development and extracellular structure organization, whereas positively enriched pathways were mainly related to neuronal maturation and synaptic function, including ligand-gated ion channel signaling, glutamate receptor and neuropeptide signaling, modulation of chemical synaptic transmission, vesicle-mediated transport, synaptic vesicle cycle and cholinergic synaptic transmission (Figure 1C). In contrast, GSEA of the P30 vs. P15 comparison identified only a single significantly enriched downregulated biological process, suggesting that the major transcriptomic transition occurs during the earlier developmental interval (Figure 1D).

We next performed Gene Ontology (GO) overrepresentation analysis to further characterize the biological functions represented by the DEGs. Between P10 and P15 the most significantly enriched biological processes for the downregulated DEGs were associated with proliferation, migration and apoptosis, whereas the ones for the upregulated DEGs were predominantly related to synaptic transmission and synaptic plasticity (Figure 1E). Consistent with the GSEA results, GO analysis for the P30 vs. P15 comparison identified few significantly enriched biological processes. Interestingly, although the upregulated DEGs were mainly associated with chromatin organization, transcription and translation (Supplementary Figure 1A), analysis of the 40 genes commonly upregulated in both developmental comparisons revealed that the five most significantly enriched biological processes were related to ion transmembrane transport (potassium in particular) and regulation of membrane potential (Figure 1F). In contrast, little significant GO enrichment was detected among either the downregulated P30 vs. P15 DEGs (Supplementary Figure 1B) or the 44 genes commonly downregulated in both developmental comparisons (Supplementary Figure 1C).

Although pathway enrichment analysis indicated that the largest developmental transition occurred between P10 and P15, inspection of the individual DEGs revealed that numerous genes associated with neuronal maturation continued to change their expression beyond P15. Furthermore, analysis focused on biologically relevant neuronal GO terms confirmed significant enrichment in processes related to synapse organization, synaptic signaling and neuronal communication among the upregulated DEGs in both developmental comparisons (Supplementary Figures 1D,E).

To visualize the developmental trajectories of representative genes involved in these biological processes, we selected DEGs from predefined, functionally curated gene sets encompassing neurogenesis (*Foxg1*, *Plxna1*, *Cux1*, *Sox11*, *Sox4*, *Meis2*, *Sema3d*, *Plxnb2*, *Cx3cr1*, *Notch2*, *Cxcl12*, *Notch1*, *Meis1*), apoptosis (*Bbc3*, *Bax*, *Bcl7c*, *Bcl2l2*, *Bad*, *Bcl9l*, *Bcl2l12*, *Tnfrsf1a*, *Bcl3*, *Tnfrsf23*, *Nod1*), synaptic signaling (Inhibitory synaptic transmission: *Gabrg2*, *Abat*, *Gabra1*, *Gabrb3*, *Gabrb1*, *Gad2*, *Gad1*, *Gabra2*, *Gabrg1*, *Gabrb2*; Excitatory synaptic transmission: *Grin2b*, *Nrxn1*, *Grin2a*, *Gria1*, *App*, *Grin2c*, *Grik5*, *Glul*, *Grik1*, *Homer1*, *Homer2*) and ion channel function (Voltage-gated potassium channels: *Kcna2*, *Kcnab3*, *Kcnab2*, *Kcnb1*, *Kcnc2, Kcnc3*, *Kcnh1*, *Kcnh7;* Voltage-gated sodium channels: *Scn1a*, *Scn3a, Scn8a, Scn4b*; Calcium channels: *Cacna1d*, *Cacna2d2*, *Trpc5, Trpc6*; Other ion channels: *Tmc7*, *Kcnj1*, *Clcn2*, *Kcnj3*, *Kcnj9*, *Kcnk1*, *Kcnma1*, *Hcn1*, *Nalcn*, *Kcnt1, Ano3*, *Cnnm1*, *Ano6*) (see also section “Materials and methods” and Supplementary Table 1). Their expression profiles were subsequently examined across the three developmental stages (Figure 1G). Genes associated with neurogenesis, forebrain development and apoptosis were markedly downregulated by P15, and this reduction was maintained at P30. In contrast, the genes associated with excitatory and inhibitory synaptic transmission were upregulated at P15, and in most cases their expression continued to increase at P30 (Figure 1G). A similar progressive transcriptional trajectory was also observed for genes encoding ion channels, in particular, voltage-gated potassium and sodium channels, as well as calcium channels and proteins involved in calcium homeostasis, highlighting the continued maturation of the molecular machinery underlying neuronal excitability beyond the first postnatal week and through the end of the first postnatal month (Figure 1G and Supplementary Table 2).

### Investigating the electrophysiological profile of SST+ cINs during later postnatal maturation

The developmental changes in ion channel expression likely manifest as corresponding changes in SST+ intrinsic membrane properties. To characterize the SST+ electrophysiological maturation, we crossed the SST-Cre driver with the Rosa26-EYFP reporter line (Srinivas et al., 2001) and performed whole-cell current-clamp recordings from YFP labeled SST+ interneurons, in acute neocortical slices, at P15 and P30. We studied passive membrane properties [the resting membrane potential (RMP) and input resistance (R_*in*_) Table 1] and active membrane properties [action potential (AP) threshold, amplitude, half-width AP rate of rise (dv/dt) Table 1] of fluorescently labeled SST+ INs. Regarding the passive properties, we observed a statistically significant decrease in the R_*in*_ between P15 and P30 (Figures 2A,C and Table 1). No significant changes were observed in AP amplitude, AP rate of rise or AP half-width (Table 1). This decrease in the R_*in*_ may be mediated in part by developmental upregulation of potassium leak channels (Goldstein et al., 2001). Indeed, our transcriptomic analysis revealed that *Kcnk3* transcript, which encodes the two-pore K+ leak channel *Task1*, is significantly enriched in P30 SST+ interneurons compared to those isolated at P15 (log_2_FC = 1.6, P30 vs. P15; Supplementary Table 2).

**TABLE 1:** Intrinsic properties of SST+ neurons recorded at P15 and P30.

Age	RMP (mV)	IR (MΩ)	AP threshold (mV)	AP amplitude (mV)	AP dv/dt (mV/ms)	AP half-width (ms)
P15	−66 ± 2	250 ± 25	−43 ± 1.5	69.2 ± 8	102 ± 23	2.3 ± 0.5
P30	−65 ± 1.2	148 ± 17*	−52 ± 3**	70 ± 3.5	120 ± 26	2.1 ± 0.3

*P < 0.05, **P < 0.01.

**FIGURE 2:**
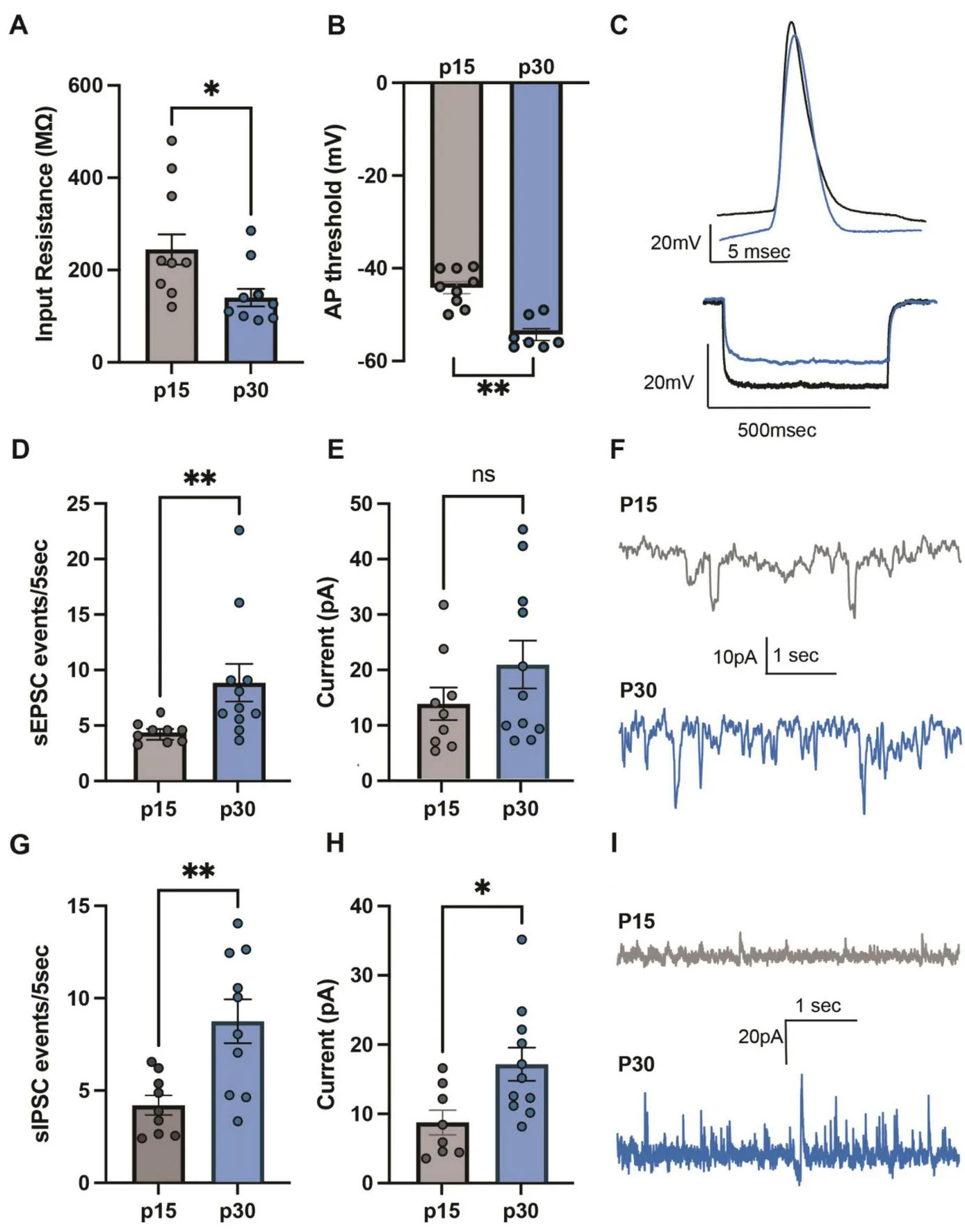
Neuronal excitability and spontaneous synaptic activity in SST+ neurons at P15 and P30. **(A,B)** The input resistance and the AP threshold of SST+ neurons were significantly decreased at P30 compared to P15 [*n* = 9 cells/ *n* = 6 animals, *t*-test, *p* = 0.01 **(A)**, *p* = 0.003 **(B)**]. **(C)** Representative traces for input resistance and action potential waveform of SST+ neurons. **(D)** The sEPSC frequency is significantly increased at P30, compared to P15 (*t*-test, *p* = 0.02). **(E)** The sEPSC amplitude is not significantly different between P15 and P30 SST+ neurons (*t*-test, *p* = 0.3). **(F)** Representative traces showing sEPSCs at P15 (top) and P30 (bottom). **(G)** The sIPSC frequency is significantly increased at P30, compared to P15 (*t*-test, *p* = 0.002). **(H)** The sIPSC amplitude is significantly increased at P30, compared to P15 (*t*-test, *p* = 0.02). **(I).** Representative traces showing sIPSCs at P15 (top) and P30 (bottom). Data for sEPSCs and sIPSCs were acquired from *n* = 9 cells/*n* = 5 animals for P15 and *n* = 11 cells/*n* = 6 animals for the P30 age group. *p < 0.05, **p < 0.01.

Concerning the action potential properties, our results revealed a significant reduction in the AP threshold of P30 SST+ interneurons, compared to the P15 ones (Figures 2B,C and Table 1). This decrease in AP threshold may be mediated in part by the upregulation of the *Scn1a* transcript (log_2_FC = 2.04, P30 vs. P15; Supplementary Table 1), which encodes the Nav1.1 voltage-gated sodium channel α-subunit (Catterall, 2010). Nav1.1 is particularly important for the firing of GABAergic interneurons and decreased levels of Nav1.1 expression, as in the case of Scn1a haploinsufficiency, lead to increased threshold for AP generation in SST+ interneurons (Tai et al., 2014).

To further support the correlation between the changes in ion channel expression levels that were indicated by our transcriptomic analysis and the electrophysiological properties we studied, we used a compartmental neuron model of an SST-like regular spiking neuron that was previously generated (Konstantoudaki et al., 2014). First, we adjusted the biophysical properties of the previously published SST neuron model to validate the electrophysiological properties of P15 SST+ neurons. Then we made the following changes in order to produce a validated P30 SST neuron model: (a) we increased the conductance of the fast-activating sodium current in accordance with increased levels of *Scn1a* expression (Table 2), (b) we increased the conductance of the delayed-rectifier potassium current, as well as, the fast-inactivating (A-type) potassium current in accordance with the increased levels of expression of *Kcna1*, *Kcnb1* and *Kcnc3* genes (log_2_FC *Kcna1* = 3.6, *Kcnc3* = 2.9, *Kcnb1* = 1.59, P30 vs. P15, Table 2 and Supplementary Table 2) and (c) we increased the conductance of the leak conductance in accordance with increased expression of *Kcnk3* (log_2_FC *Kcnk3* = 1.6, P30 vs. P15, Table 2 and Supplementary Table 2). Our neuron model with the above adjustments validated the recorded properties of SST+ neurons at P15 and P30.

**TABLE 2:** Conductances of key biophysical properties of the P15 and P30 SST+ neuron model.

Current	Compartments	P30 model conductance (S/cm^2^)	P15 model conductance (S/cm^2^)
Leak conductance	Soma, dend, axon	9e-5	6e-5
Voltage-gated sodium current	Soma, dend, axon	2.25e-2	1.35e-2
Delayed-rectifier potassium current	Soma, dend, axon	1.44e-1	9e-3
Slowly inactivating potassium current	Soma	7.25e-4	3.625e-5
A-type potassium current	Soma, dend	7e-4	7e-5
Calcium-dependent potassium current	soma	1e-6	1e-7
L-type calcium current	soma	1e-3	1e-3
T-type potassium current	soma	3e-3	3e-3

Finally, we measured developmental changes in excitatory and inhibitory synaptic input to fluorescently labeled SST+ interneurons by recording spontaneous excitatory post-synaptic currents (sEPSCs), while holding the membrane potential at −60 mV, and spontaneous inhibitory post-synaptic currents (sIPSCs), while holding the membrane potential at +10mV. We measured the frequency and amplitude of sEPSCs and sIPSCs. We found that both sEPSC and sIPSC frequency are significantly increased at P30 compared to P15 (Figures 2D,F,G,I). In addition, the amplitude of sIPSCs was also significantly increased at P30, compared to P15, while the sEPSC amplitude was not different between the two age groups (Figures 2E,H). Interestingly, these changes agree with the significant changes in the expression levels of GABA_*A*_- receptor subunits (log_2_FC *Gabrb2* = 3.08, *Gabrb1* = 1.9, *Gabrq* = −3.17, P30 vs. P15; Supplementary Table 2) and a modest increase in AMPA receptor subunits (log_2_FC *Gria3* = 0.96, *Gria2* = 0.9, P30 vs. P15; Supplementary Table 2).

## Discussion

In this work we identified changes in the whole-transcriptome expression and in the intrinsic and synaptic physiology of genetically labeled SST+ cINs during the P10-P30 developmental window. Previous studies have assayed whole-transcriptome analysis in SST+ cINs in mice over similar periods of maturation (Favuzzi et al., 2019; Stroud et al., 2020). In agreement with Stroud et al. (2020), we identified that midway through the first postnatal month there is a major switch, with genetic programs implicated in early development being downregulated and the ones related to the mature intrinsic and synaptic features of SST+ cINs upregulated. Furthermore, our work determined that this transition toward the mature transcriptomic profile of SST+ cINs occurs soon after the cell death of cINs is concluded, between P10 and P15 (Southwell et al., 2012; Wong et al., 2019). This transcriptional switch does not strictly correlate with the onset of expression of genes tied with the maturation of SST+ cINs. Several studies have shown that a variety of ion channels and synapse-related genes are already expressed in cINs -including SST+ cINs- during embryogenesis and the first postnatal week (Batista-Brito et al., 2008; Marsh et al., 2008; Favuzzi et al., 2019; Munguba et al., 2023). Our analysis reflects rather that the passage from immature to mature states -based on the transcription profile of genes implicated in neurotransmission, ion channel activity, synaptic function and plasticity- is dynamically modulated throughout the whole first postnatal month, providing evidence that the maturation process of SST+ cINs is similarly protracted compared to other cIN types (Okaty et al., 2009).

Interestingly, the late postnatal changes in ion channel expression were reflected in changes in the electrophysiological properties of SST+ cINs. Between P15 and P30 SST+ cINs showed a decline in input resistance and required smaller current injections to reach spike threshold, while the rest of the active and passive properties examined did not change markedly. Previous studies suggested that the intrinsic development of SST+ cIN passive properties lasts until the second postnatal week -when the resting membrane potential increases and the input resistance decreases but plateaued after P15- while the active properties of interneurons, including action potential kinetics, show sustainable growth (Kinnischtzke et al., 2012; Pan et al., 2016, 2019). Overall, our results agree with the above observations, except for input resistance which we showed is further decreased after P15. This could reflect differences among diverse SST+ cIN types and/or their topography -in terms of layer acquisition and/or brain region- as they are integrating into distinct developing cortical circuits. In our studies we used the SST-CRE driver that labels all SST+ INs (Taniguchi et al., 2011) and we analyzed SST+ cINs located in all cortical layers, while the above studies have used the GIN transgenic line that primarily labels Martinotti cINs (Oliva et al., 2000; Ma et al., 2006) and examined the electrophysiological properties of superficial (layer II/III) SST+ neurons. Such experimental modifications may explain the variations in the developmental trajectory of intrinsic properties of SST+ cINs we observed compared to previously published work (Lazarus et al., 2011; Kinnischtzke et al., 2012; Pan et al., 2016, 2019).

Although we did not directly explore mechanisms underlying this process, our transcriptomic analysis revealed significant developmental regulation of multiple ion channel genes (FDR < 0.05). Notably, several voltage-gated potassium channel subunits were strongly upregulated at P30, including *Kcna1*, *Kcnc3*, *Kcnh7*, and *Kcnc1*, as well as *Kcnb1* and *Kcna2*. In addition, we observed increased expression of calcium-activated potassium channel components such as *Kcnma1* and its auxiliary subunit *Kcnmb2*. These changes are consistent with enhanced potassium conductance that could contribute to the reduced input resistance observed at P30 (Rudy and McBain, 2001; Bean, 2007). We also detected upregulation of leak and inwardly rectifying potassium channels, including *Kcnk3* and *Kcnj9*, further supporting a shift toward increased membrane conductance in mature SST+ interneurons (Goldstein et al., 2001; Enyedi and Czirják, 2010). In contrast, a small subset of potassium channel-related genes was downregulated, including *Kcnk9*, *Kctd2*, and *Kcnip1*, suggesting selective remodeling rather than uniform upregulation of all K^+^ channel subtypes. In agreement with the observed decrease in action potential threshold, we also found a robust upregulation of sodium channel genes, particularly *Scn1a*, which encodes the Nav1.1 α-subunit, as well as *Scn3a* (Catterall, 2010). Given the established role of Nav1.1 in supporting interneuron excitability, this increase likely contributes to the enhanced firing properties of SST+ interneurons at P30 (Tai et al., 2014). Overall, these transcriptomic changes indicate a coordinated upregulation of sodium, potassium, and calcium conductances, supporting the maturation of intrinsic excitability and membrane properties in SST+ interneurons between P15 and P30 (Moody and Bosma, 2005; Spitzer, 2006).

In addition to changes in intrinsic physiological properties, we also observed dynamic regulation of synaptic properties during the last half of the first postnatal month. The frequency of both sEPSCs and sIPSCs increased between P15 and P30, while only the amplitude of sIPSCs showed an overall increase during the same developmental period, in agreement with previously published work (Kinnischtzke et al., 2012; Pan et al., 2016, 2019). Consistent with the most prominent changes in sIPSCs amplitude, our transcriptomic analysis detected an increase in the ratio of *Gabrb2* expression to *Gabrb1, Gabrb3* and *Gabrq* expression levels -with the last one being more dramatic- indicative of SST+ cIN’s transition from immature θ-driven slow, diffuse inhibition to mature β2-driven fast, more precise synaptic inhibition, while retaining β1 and β3 expression that preserves their role in long-timescale dendritic control (Laurie et al., 1992; Poulter et al., 1999; Farrant and Nusser, 2005; Lüscher et al., 2011). Interestingly, *Gabrα1, Gabra2* and *Gabrg2* that encode for three fundamental GABA_*A*_-receptor subunits, with the γ2 subunit having an established role in maintaining receptors at mature inhibitory synapses (Schweizer et al., 2003), were significantly upregulated only between P15 and P10. This temporal distinct regulation of GABA_*A*_-receptor subunits reflects distinct steps during the maturation of inhibitory synapses, with the establishment of key components associated with mature GABA_*A*_ receptors during the second postnatal week, followed by subunit refinement and further maturation of synaptic connectivity onto SST+ cINs up to P30 (Zavalin et al., 2024). Finally, it is worth mentioning that we did not observe significant changes in the expression levels of genes encoding chloride transporters, such as *Slc12a5* and *Slc12a2*. This likely reflects the fact that major developmental changes in neuronal chloride handling occur predominantly before the beginning of the developmental window examined here (Ben-Ari, 2002).

Regarding the increased frequency of sEPSCs, this could be explained by the modest increase in AMPA receptor subunits Gria2 and Gria3, as well as by the increased expression of genes involved in synaptic post-translational modification of AMPA receptor subunits. Similar results were observed for both superficial SST+ interneurons (Pan et al., 2016) and pyramidal neurons (Burnsed et al., 2023).

Despite providing a comprehensive characterization of SST+ cIN maturation, several limitations should be considered. First, our transcriptomic analysis was performed using bulk RNA sequencing, which may obscure subtype-specific, layer-specific or region-specific transcriptional differences within the heterogeneous SST+ cIN population. Future single-cell and spatial transcriptomic approaches will be necessary to resolve these distinctions. Second, although we identify strong correlations between ion channel gene expression and electrophysiological properties, our study does not establish direct causal relationships. Targeted genetic or pharmacological manipulations will be required to determine the specific contribution of individual channels to the maturation of SST+ interneuron excitability. Additionally, our electrophysiological recordings were restricted to defined developmental time points and cortical regions, and further work will be needed to determine how these processes vary across circuits, behavioral states, and activity-dependent conditions.

Notwithstanding these limitations, our findings provide important conceptual advances by demonstrating that SST+ interneuron maturation is not completed by the end of the second postnatal week, as previously suggested, but instead continues through later postnatal stages. By linking sustained transcriptional changes in ion channel and synaptic genes with corresponding refinements in intrinsic and synaptic physiology, and further validating these relationships through computational modeling, this study establishes a coordinated and protracted developmental program underlying SST+ interneuron maturation. Given the critical role of SST+ interneurons in cortical computation and their involvement in neurodevelopmental and neuropsychiatric disorders, defining this extended maturation window has important implications for understanding both normal circuit assembly and the temporal vulnerability of inhibitory networks in disease.

## Data Availability

The datasets presented in this study can be found in online repositories. The names of the repository/repositories and accession number(s) can be found below: https://www.ncbi.nlm.nih.gov/geo/, GSE333340.
