# Supplementary material for: SST interneuron maturation extends beyond the second postnatal week and is driven by coordinated transcriptional and functional remodeling
Source: Front Mol Neurosci. 2026 Sep 15;19:1892850. doi: 10.3389/fnmol.2026.1892850 (PMC13619902; doi:10.3389/fnmol.2026.1892850)
Supplement: Supplementary file 1 [file Data_Sheet_1.pdf]

Heatmap Title	Gene	QIAGEN RT² Profiler PCR Array	Functional subgroup within the array
Neurogenesis	Foxg1	Mouse Neurogenesis & Neural Stem Cells	Forebrain development
	Plxna1	Mouse Neurogenesis & Neural Stem Cells	Axon guidance
	Cux1	Mouse Neurogenesis & Neural Stem Cells	Cortical neuron differentiation
	Sox11	Mouse Neurogenesis & Neural Stem Cells	Neuronal differentiation
	Sox4	Mouse Neurogenesis & Neural Stem Cells	Neuronal differentiation
	Meis2	Mouse Neurogenesis & Neural Stem Cells	Neuronal specification
	Sema3d	Mouse Neurogenesis & Neural Stem Cells	Axon guidance
	Plxnb2	Mouse Neurogenesis & Neural Stem Cells	Axon guidance
	Cx3cr1	selected from RNAseq	Neuron–glia interactions
	Notch2	Mouse Neurogenesis & Neural Stem Cells	Neural progenitor maintenance
	Cxcl12	Mouse Neurogenesis & Neural Stem Cells	Cell migration
	Notch1	Mouse Neurogenesis & Neural Stem Cells	Neural progenitor maintenance
	Meis1	Mouse Neurogenesis & Neural Stem Cells	Neural development
Apoptosis	Bbc3 (Puma)	Mouse Apoptosis	Intrinsic apoptosis
	Bax	Mouse Apoptosis	Intrinsic apoptosis
	Bcl7c	selected from RNAseq	BCL-associated protein
	Bcl2l2 (Bcl-w)	Mouse Apoptosis	Anti-apoptotic BCL2 family
	Bad	Mouse Apoptosis	Pro-apoptotic BCL2 family
	Bcl9l	selected from RNAseq	Cell survival/Wnt-associated
	Bcl2l12	Mouse Apoptosis	Anti-apoptotic BCL2 family
	Tnfrsf1a	Mouse Apoptosis	Death receptor signaling
	Bcl3	Mouse Apoptosis	NF-κB signaling
	Tnfrsf23	Mouse Apoptosis	TNF receptor family
	Nod1	Mouse Apoptosis	Death/inflammatory signaling
GABAergic synapses	Gabrg2	Mouse Synaptic Plasticity	GABA receptor
	Abat	Mouse Synaptic Plasticity	GABA metabolism
	Gabra1	Mouse Synaptic Plasticity	GABA receptor
	Gabrb3	Mouse Synaptic Plasticity	GABA receptor
	Gabrb1	Mouse Synaptic Plasticity	GABA receptor

	Gad2	Mouse Synaptic Plasticity	GABA synthesis
	Gad1	Mouse Synaptic Plasticity	GABA synthesis
	Gabra2	Mouse Synaptic Plasticity	GABA receptor
	Gabrg1	Mouse Synaptic Plasticity	GABA receptor
	Gabrb2	Mouse Synaptic Plasticity	GABA receptor
Glutamatergic synapses	Grin2b	Mouse Synaptic Plasticity	NMDA receptor
	Nrxn1	Mouse Synaptic Plasticity	Synaptic adhesion
	Grin2a	Mouse Synaptic Plasticity	NMDA receptor
	Gria1	Mouse Synaptic Plasticity	AMPA receptor
	App	Mouse Synaptic Plasticity	Synaptic function
	Grin2c	Mouse Synaptic Plasticity	NMDA receptor
	Grik5	Mouse Synaptic Plasticity	Kainate receptor
	Glul	Mouse Synaptic Plasticity	Glutamate metabolism
	Grik1	Mouse Synaptic Plasticity	Kainate receptor
	Homer1	Mouse Synaptic Plasticity	Postsynaptic scaffold
	Homer2	Mouse Synaptic Plasticity	Postsynaptic scaffold
Ion channels and ligand-gated ion channels	Kcnj3	Mouse Ion Channels & Transporters	Inward rectifier K^+ channel
	Kcnj9	Mouse Ion Channels & Transporters	Inward rectifier K^+ channel
	Kcnk1	Mouse Ion Channels & Transporters	Two-pore K^+ channel
	Kcnma1	Mouse Ion Channels & Transporters	BK potassium channel
	Hcn1	Mouse Ion Channels & Transporters	HCN channel
	Nalcn	Mouse Ion Channels & Transporters	Sodium leak channel
	Kcnt1	Mouse Ion Channels & Transporters	Na^+-activated K^+ channel
	Trpc6	Mouse Ion Channels & Transporters	TRP channel
	Ano3	Mouse Ion Channels & Transporters	Ca^{2+}-activated Cl^- channel
	Cnnm1	Mouse Ion Channels & Transporters	Magnesium transporter
	Ano6	Mouse Ion Channels & Transporters	Ca^{2+}-activated Cl^- channel
	Trpc5	Mouse Ion Channels & Transporters	TRP channel
	Tmc7	Mouse Ion Channels & Transporters	Membrane channel protein
Voltage-gated ion channels	Kcna2	Mouse Ion Channels & Transporters	Voltage-gated K^+ channel

	Kcnab3	Mouse Ion Channels & Transporters	K^+ channel β subunit
	Kcnab2	Mouse Ion Channels & Transporters	K^+ channel β subunit
	Kcnb1	Mouse Ion Channels & Transporters	Voltage-gated K^+ channel
	Scn1a	Mouse Ion Channels & Transporters	Voltage-gated Na^+ channel
	Scn3a	Mouse Ion Channels & Transporters	Voltage-gated Na^+ channel
	Cacna1d	Mouse Ion Channels & Transporters	L-type Ca^{2+} channel
	Kcnj1	Mouse Ion Channels & Transporters	Inward rectifier K^+ channel
	Kcnc3	Mouse Ion Channels & Transporters	Voltage-gated K^+ channel
	Kcnh1	Mouse Ion Channels & Transporters	Voltage-gated K^+ channel
	Cacna2d2	Mouse Ion Channels & Transporters	Ca^{2+} channel auxiliary subunit
	Kcnb1	Mouse Ion Channels & Transporters	Voltage-gated K^+ channel
	Kcnc2	Mouse Ion Channels & Transporters	Voltage-gated K^+ channel
	Scn8a	Mouse Ion Channels & Transporters	Voltage-gated Na^+ channel
	Kcnc1	Mouse Ion Channels & Transporters	Voltage-gated K^+ channel
	Clcn2	Mouse Ion Channels & Transporters	Chloride channel
	Kcnh7	Mouse Ion Channels & Transporters	Voltage-gated K^+ channel
	Scn4b	Mouse Ion Channels & Transporters	Sodium channel β subunit

Supplementary Table 1. Table of genes included in the heatmaps of Figure 1.
