# Supplementary material for: SST interneuron maturation extends beyond the second postnatal week and is driven by coordinated transcriptional and functional remodeling
Source: Front Mol Neurosci. 2026 Sep 15;19:1892850. doi: 10.3389/fnmol.2026.1892850 (PMC13619902; doi:10.3389/fnmol.2026.1892850)
Supplement: Supplementary file 2 [file Data_Sheet_2.pdf]

**P15 vs P10**			**P30 vs P15**		
	FDR	log_2_FC>1		FDR	log_2_FC>1
**Potassium Channels**					
Kcna2	1.72E-05	1.72900887	*Kcna1*	8.33E-17	3.633944306
Kcnc1	7.16E-09	1.71584598	*Kcnc3*	4.09E-07	2.903138461
Kcnj9	0.000879	1.65367525	*Kcnh7*	0.00293	2.309046804
Kcnh1	0.013781	1.60334103	*Kcnmb2*	7.76E-09	1.813121865
Kctd8	0.000369	1.59693514	*Kcnh1*	1.73E-05	1.603109847
Kcnt1	0.033775	1.46844849	*Kcnk3*	0.006553	1.604435457
Kcnma1	3.68E-05	1.43757604	*Kcnb1*	4.05E-08	1.592033216
Kcnab1	0.006678	1.3734584	*Kcnma1*	3.92E-09	1.540760421
Kcnab3	0.000445	1.28136105	*Kcnc1*	4.25E-07	1.409814638
Kcnab2	0.00258	1.2735014	*Kctd16*	0.015959	1.304479124
Kcnj3	0.018891	1.25375659	*Kcna2*	0.000301	1.226117911
Kcnf1	0.032732	1.16425625	*Kcnj9*	0.004228	1.062914207
Kcnc2	0.022352	1.06927571	*Hcn1*	2.40E-06	1.068531382
Kcnj4	0.028077	1.03876957	*Kcnip1*	0.018433	-1.02832527
Hcn1	7.05E-07	1.60064412	*Kctd2*	4.67E-05	-1.26954662
Kctd16	0.024154	-1.21222062	*Kcnk9*	0.011271	-1.48795618
Kctd15	0.001875	-3.55458885			
**Sodium Channels**					
Scn4b	0.001233	5.10852446	*Scn1a*	2.94E-07	2.042749647
Scn1a	0.000455	2.03538288	*Scn3a*	0.008063	1.277533976
Scn2b	1.58E-09	1.96259201			
Scn8a	0.001768	1.05123263			
Scnm1	0.0447	-1.52714787			
**Glutamate receptors**					
	FDR<0.05	log_2_FC>0.5		FDR<0.05	log_2_FC>0.5
Gria1	3.07E-06	1.20112473	*Gria2*	0.019552	0.901159396
Gria2	0.007284	1.01813822	*Gria3*	0.030908	0.968705729
Gria4	0.029986	0.96168307	*Grin2a*	2.95E-05	1.198494154
Gria3	0.034424	0.94263378	*Grin2b*	6.09E-05	1.610987018
Grik5	0.041653	1.06428483	*Grm1*	0.028132	0.877143252
Grik3	0.014255	0.8674839	*Grin2d*	0.000491	-1.6596973
Grik1	0.012224	1.39635294	*Grin1*	0.026541	-0.80710448
Grin2a	1.82E-07	2	*Grm4*	0.013674	-1.04784142
Grm1	0.036165	0.89068331			
**Gaba receptors**					
	FDR<0.05	log_2_FC>0.5		FDR<0.05	log_2_FC>0.5
Gabra1	3.00E-09	2.34050952	*Gabra1*	4.49E-06	0.657262378
Gabra2	0.018222	1.20824193	*Gabrb1*	4.49E-06	1.966031064
Gabrb3	0.001472	1.03575233	*Gabrb2*	0.030673	3.078002512
Gabra3	0.015678	-1.43850143	*Gabrq*	0.001413	-3.169925

Supplementary Table 2. Table of selected differentially expressed genes (DEGs) in the SST^+ cIN population [false discovery rate (FDR) threshold of 0.05 and absolute fold change (FC) cutoff value of 1.0 or 0.5 in $\log_2$ scale] for both P15 versus P10 and P30 versus P15 comparisons. Common DEGs for both comparisons are labelled blue. Only one gene (*Kctd16*, in purple) shows opposite expression profiles between the two comparisons. For each DEG upregulated FCs are shown in green and downregulated FCs are shown in red. Notice that the majority of DEGs for the selected categories (ion channels, glutamate and GABA receptors) are upregulated during the developmental periods examined.
