# Supplementary material for: SST interneuron maturation extends beyond the second postnatal week and is driven by coordinated transcriptional and functional remodeling
Source: Front Mol Neurosci. 2026 Sep 15;19:1892850. doi: 10.3389/fnmol.2026.1892850 (PMC13619902; doi:10.3389/fnmol.2026.1892850)
Supplement: Supplementary file 3 [file Supplementary_file_1.docx]

**Supplementary Figure 1**

**Supplementary Figure 1.**

**(A, B)** Gene Ontology (GO) Biological Process over-representation analysis of DEGs (FDR≤0.05 and │log2 fold change│≥1) identified in the P30 vs P15 comparison. **(A)** Representative biological processes most significantly enriched among upregulated genes. **(B)** Representative biological processes most significantly enriched among downregulated genes. Bars represent -log10 (adjusted P value). Only biological terms with -log10 (adjusted P value) > 1.3 corresponding to adjusted p<0.05 are considered statistically significant **(C)** Top, Venn diagram showing overlap between genes downregulated in the P15 vs P10 and the P30 vs P15 comparisons. Bottom, GO Biological Process over-representation analysis of the 44 commonly downregulated genes. **(D, E)** Representative biologically relevant neuronal GO terms significantly enriched among upregulated genes in the P15 vs P10 **(D)** and P30 vs P15 **(E)** comparisons.
